# Bridging Social Networks and Well-Being: The Moderating Power of Technology Use in Older Adults

**DOI:** 10.1093/geroni/igaf122.2794

**Published:** 2025-12-31

**Authors:** Manjun Kim, Yesang Cho

**Affiliations:** Florida State University, Tallahassee, Florida, United States; Seoul National University, Seoul, Republic of Korea

## Abstract

Social networks are widely recognized as essential to the well-being of older adults. As technology continues to advance, its use has also become important in forming and maintaining social connections. Particularly, technology use is crucial in supporting older adults and shaping their social engagement(Kim et al., 2017). While technology adoption rates among older adults have steadily increased over time (Mitzner et al., 2019), consideration must be given to how it integrates with beneficial and related factors. Within the framework of Socioemotional Selectivity Theory (SST), older adults prioritize meaningful relationships and prioritize emotionally fulfilling experiences (Carstensen et al., 1999). Technology use enhances older adults’ social networking, leading to improved well-being (Baradach et al., 2021; Fang et al., 2018). This study further explores how information and communication technology (ICT)_ use impacts their well-being. Using National Health and Aging Trends Study Round 13, we employed a hierarchical multi-regression model to analyze how ICT use moderates the relationship between social network and well-being. The results indicate that ICT use moderated the relationship between social networks and well-being This effect was pronounced among individuals with lower social networking. Our findings suggest that ICT use may serve as a protective factor against declines in well-being among older adults with lower levels of social connections. Specifically, ICT users who had a smaller well-being gap associated with differences in social network size compared to non-users. These results highlight the significance of technology adoption in later life and underscore the potential of technology use to mitigate disparities in well-being.

